# Network-based multi-class classifier to identify optimized gene networks for acute leukemia cell line classification

**DOI:** 10.1371/journal.pone.0321549

**Published:** 2025-05-08

**Authors:** Heewon Park, Satoru Miyano

**Affiliations:** 1 School of Mathematics, Statistics and Data Science, Sungshin Women’s University, Seoul, Republic of Korea; 2 M&D Data Science Center, Tokyo Medical and Dental University, 1-5-45 Yushima, Bunkyo-ku, Tokyo, Japan; 3 Human Genome Center, The Institute of Medical Science, The University of Tokyo, 4-6-1 Shirokane-dai, Minato-ku, Tokyo, Japan; University of Missouri, UNITED STATES OF AMERICA

## Abstract

Unraveling the genetic regulatory networks that underlie diseases is essential for comprehending the intricate mechanisms of these conditions. While various computational strategies were developed, the approaches in the existing studies concerning network-based prediction and classification are based on the pre-estimated gene networks. However, the gene network that is pre-estimated fails to yield biologically meaningful explanations for classifying cell lines into particular clinical states. The reason for this limitation is the lack of inclusion of any information about the clinical status of cell lines during the process of network estimation. To achieve effective cell line classification and ensure the biological validity of the cell lines classification, we develop a computational strategy referred to as GRN-multiClassifier for network-based multi-class classification. The GRN-multiClassifier estimates gene network in a manner that simultaneously minimizes both the network estimation error and the negative log-likelihood function of multinomial logistic regression. That is, our strategy estimates optimized gene network to enable the multi-class classification of cell lines into specific clinical conditions. Monte Carlo simulations demonstrate the efficacy of the GRN-multiClassifier. We applied our strategy to network-based classification of acute leukemia cell lines into three distinct categories of acute leukemia. Our strategy shows outstanding performance in the classification of acute leukemia cell lines. The results for the acute leukemia marker identification are strongly supported by existing literature. The implications of our findings suggest that potential pathways involving the inhibition of ACTB and the molecular interactions between “HBA1&HBB,” “HBB&HBA1,” “IGKV1-5&IGHV4-31,” “IGHV4-31&IGKV1-5,” “HLA-DRA&CD74” and “ACTB&ACTB” could offer significant insights into the underlying mechanism of acute leukemia.

## Introduction

Gene expression level analysis alone is insufficient to comprehend the intricate mechanisms driving certain disease states, as diseases involved in complex mechanism arise from disturbances in the specific operations of molecular networks rather than anomalies in individual genes [1]. In the field of biomedical research, one of the most impactful technique is heterogeneous gene regulatory network analysis and the study of gene networks has gained significant research attention in various fields of research. The effectiveness of network-based analysis has been validated in various research works, e.g., cancer prediction, drug combinations identification, and protein-protein interaction [2–4]. In particular, various investigations have delved into network-based prediction and classification, and their effectiveness has been verified [5, 6]. Although various computational methods have been developed and employed to predict and/or classify the cancer-related status of cell lines, previous studies performed the gene network analysis based on the pre-estimated gene networks. In other words, the existing studies estimated gene networks in advance based on gaussian graphical modeling, Bayesian network, etc. [7, 8], and then used the pre-estimated gene network to classification, prediction, uncovering disease-related mechanisms. However, the analysis conducted using pre-estimated gene networks lacks the capacity to provide biologically reliable results, as it does not incorporate crucial biological mechanisms, such as those related to cancer, into the network estimation process.

In order to achieve biologically reliable results of classification and gene network analysis, we develop computational approach termed Gene Regulatory Network-based multi-class Classifier (GRN-multiClassifier), designed for gene network-based multi-class classification, following the methodology proposed by Park *et al*. [9]. The objective function of the GRN-multiClassifier is based on the negative log-likelihood function for the multinomial logistic regression model and error term of gene network estimation. A crucial aspect to highlight is that our approach conducts gene network estimation and multi-class classification simultaneously. In our strategy, the input of multi-class classification model is the gene network that is estimated to minimize both the error in network estimation and the negative log-likelihood function, simultaneously. That is, the gene network estimation is concurrently performed with cell line classification by minimizing not only network estimation error but also classification error. It implies that the estimated gene networks are iteratively updated to optimize multi-class classification. The optimized gene networks may have crucial information to characterize each class and it can be useful tool to identify markers of specific phenotypes. Thus, we can efficiently perform for biologically reliable interpretation of the results arising from gene network estimation and the classification of cell lines. On the other hand, pre-estimated networks are estimated by minimizing only the network estimation error without consideration of classification. Thus, the pre-estimated networks cannot describe the optimized molecular interplays to classify the cell lines.

Through Monte Carlo simulations, we show the effectiveness of the GRN-multiClassifier in both multi-class classification and gene network estimation. We applied the GRN-multiClassifier to the acute leukemia gene expression dataset comprising 72 cell lines and 7129 genes [10], facilitating gene network-based classification of acute leukemia cell lines. Our strategy shows effective performance in the classification of cell lines associated with acute leukemia. We then identify the markers relevant to acute leukemia as well as their associated molecular interplays and the validity of the identified markers is confirmed through existing literature. While biological knowledge was not incorporated into the identfied markers and their networks, our data-driven strategy yields biologically reliable outcomes for the identification of acute leukemia markers. The results of our study suggest that the increased expression of ACTB and the molecular interplays involving “HBA1&HBB," “HBB&HBA1," “IGKV1-5&IGHV4-31," “IGHV4-31&IGKV1-5," “HLA-DRA&CD74" and “ACTB&ACTB" might contribute to the progression of acute leukemia. In light of this, we suggest that the suppression of ACTB and the associated molecular interactions could provide vital insights into understanding potential strategies for both preventing and treating acute leukemia.

The remainder of this paper is organized as follows: In the Methods section, we outline the computational methods for gene network estimation and as well as the novel strategy we have developed for network-based multi-class classification. Subsequently, we introduce the numerical resolution process for the GRN-multiClassifier. In the section of Monte Carlo simulation, we demonstrate the results derived from the executed simulation studies. The findings of gene network-based acute leukemia cell line classification are detailed in the section titled Gene Regulatory Network-based Acute Leukemia Subtypes Classification. Concluding remarks and insights are presented in the Discussion section.

## Methods

### Gene regulatory network estimation

Suppose $X=(x_{1},...,x_{n}{)}^{T}\in {\mathbb{R}}^{n\times p}$ is an n×p data matrix describing the expression levels of *p* regulator genes that control the ${\text{j}}^{\text{th}}$ target gene transcription $y_{j}\in {\mathbb{R}}^{n}$, j=1,...,k.

The gene regulatory network can be represented by the following linear regression model,

$$y_{ij}=\beta_{j}^{T}x_{i}+\epsilon_{ij},\;i=1,...,n,\;j=1,...,k,$$ (1)

where $\beta_{j}=(\beta_{j1},...,\beta_{jp}{)}^{T}$ is the regression coefficient that represents the effect of *p* regulator genes $x_{i}$ on ${\text{j}}^{\text{th}}$ target gene *y*_*ij*_ and $\epsilon_{ij}$ is a random error vector for the ${\text{j}}^{\text{th}}$ target gene. The regularization methods have been often used to estimate the gene networks,

$$\underset{\beta_{j}}{\text{arg\textasciitilde{}min}}\{\sum_{i=1}^{n}(y_{ij}-\beta L_{j}^{T}x_{i}{)}^{2}+P(\beta_{j})\},$$ (2)

where $P(\beta_{j})$ is the regularization penalty, e.g., ridge [11], lasso [12], elastic net [13], etc.,

$$P(\beta_{j})=\frac{\lambda_{1}}{2}\sum_{\ell =1}^{p}\beta_{j\ell }^{2}+\lambda_{2}\sum_{\ell =1}^{p}|\beta_{j\ell }|,$$ (3)

and $\lambda_{1},\lambda_{2}>0$ are the regularization parameters of $\beta_{j}$.

The gene networks are crucial to understand complex cancer-related mechanisms, because the mechanisms are involved in dysregulation and dysfunction of molecular networks. Although numerous computational methodologies have been formulated and applied to gene network analysis, the methods cannot provide biologically reliable interpretation of the results. This limitation arises from the fact that the process of estimating gene networks did not consider cancer-associated mechanisms, i.e., the networks are pre-determined, and these pre-estimated gene networks are subsequently applied for tasks like classification, prediction, and the exploration of disease-related mechanisms. In order to identify molecular interplays that are optimized to explain cancer-related status of cell lines (e.g., clones), we developed a novel computational approach named Gene Regulatory Network based multi-class Classifier (GRN-multiClassifier).

### Preliminaries

Let *Z*_*i*_ is categorical response variable having *G*>2 levels to describe phenotypes of ${\text{i}}^{\text{th}}$ cell line and $x_{i}$ is expression of *p* gene in ${\text{i}}^{\text{th}}$ cell line. Suppose we have *n* independent observations $\left\{(Z_{i},x_{i}),i=1,...,n\right\}$, where *Z*_*i*_ is categorical response variable having *G*>2 levels. The multinomial logistic regression model for multi-class classification assumes that

$$\text{Pr}(Z_{i}=g|x_{i})=\pi_{g}(x_{i})=\frac{\text{exp}(\theta_{g0}+x_{i}^{T}\theta_{g})}{\sum_{g=1}^{G}\text{exp}(\theta_{g0}+x_{i}^{T}\theta_{g})}\;\text{or}\;\text{log}\frac{\pi_{g}(x_{i})}{\pi_{G}(x_{i})}=\theta_{g0}+x_{i}^{T}\theta_{g},$$ (4)

where $x_{i}=(x_{i1},...,x_{ip}{)}^{T}$. We suppose the ${\text{g}}^{\text{th}}$ element in ${\text{i}}^{\text{th}}$ row of $Z\in {\mathbb{R}}^{n\times G}$ equals one when the ${\text{i}}^{\text{th}}$ cell line belongs to phenotype *g*, and zero otherwise, i.e., $z_{i}=(z_{i1},...,z_{iG})$ represents the multinormial trial for subject *i* with *z*_*ig*_ = 1 when the response is in phenotype *g* and *z*_*ig*_ = 0 otherwise. In other words, $z_{i}$ serves to phenotypes of cell lines and is a random variable with multinormial distribution, such that $z_{i}\sim \text{Multinomial}(\pi_{1}(x_{i}),...,\pi_{G}(x_{i}))$, having the following probability mass function,

$$f(z_{i}|x_{i};\theta )=\frac{1}{z_{i1}!\cdots z_{iG}!}\prod_{g=1}^{G}\pi_{g}(x_{i}{)}^{z_{ig}},$$ (5)

where $\sum_{g=1}^{G}\pi_{g}(x_{i})=1$. The log-likelihood has the follows form


$$\ell \ell (\Theta )=\text{log}[\prod_{i=1}^{n}(\frac{1}{z_{i1}!\cdots z_{iG}!}\prod_{g=1}^{G}\pi_{g}(x_{i}{)}^{z_{ig}})]$$

$$≃\sum_{i=1}^{n}(\sum_{g=1}^{G}z_{ig}(\theta_{g0}+x_{i}^{T}\theta_{g})-\text{log}\{\sum_{g=1}^{G}\text{exp}(\theta_{g0}+x_{i}^{T}\theta )\}),$$ (6)

where $\Theta =(\theta_{10},...,\theta_{G0},\theta_{1}^{T},...,\theta_{G}^{T}{)}^{T}$. Although maximum likelihood method has been used to estimate the multinormial logistic regression model, the method frequently yields unstable estimation results with significant variation. This instability is particularly prominent when multicollinearity exists among predictor variables or when dealing with datasets of high dimensionality [14]. To address the issue, we consider the following penalized log likelihood methods,

$$\underset{\Theta }{\text{arg\textasciitilde{}min}}[-\sum_{i=1}^{n}(\sum_{g=1}^{G}z_{ig}(\theta_{g0}+x_{i}^{T}\theta_{g})-\text{log}[\sum_{g}^{G}\text{exp}(\theta_{g0}+x_{i}^{T}\theta_{g})])\;\;+\lambda_{1}\sum_{g}^{G}\sum_{\ell =1}^{p}|\theta_{g\ell }|+\frac{\lambda_{2}}{2}\sum_{g}^{G}\sum_{\ell =1}^{p}\theta_{g\ell }^{2}]$$ (7)

Most of the previous studies in the domain of multi-class classification for cell lines were based on the gene expression levels as the input for the multinomial logistic regression model, i.e., the input of the multinormial logistic regression model was expression levels of genes X. However, a single gene-based analysis is insufficient in generating biologically reliable results and meaningful interpretations for the classification of cell lines. This is due to the fact that the complex mechanisms of diseases are intricately linked to molecular networks, rather than being solely reliant on the perturbation of individual genes.

### Gene regulatory network based multi-class classifier: GRN-multiClssifier

We consider the network-based multi-class classification and develop a computational strategy that performs network estimation and classification, simultaneously. For the categorical response variable *Z*_*i*_ describing phenotypes of cell lines, the network-based multi-class classification is performed by the expression levels of genes $X=(x_{1},...,x_{n}{)}^{T}$ and their regulatory networks $B=(\beta_{1},...,\beta_{k})\in {\mathbb{R}}^{p\times k}$ as follows,

$$\text{Pr}(Z_{i}=g)=\pi_{g}(x_{i},B)=\frac{\text{exp}(\theta_{g0}+x_{i}^{T}B\theta_{g})}{\sum_{g=1}^{G}\text{exp}(\theta_{g0}+x_{i}^{T}B\theta_{g})}$$ (8)

$$\text{log}\frac{\pi_{g}(x_{i},B)}{\pi_{G}(x_{i},B)}=\theta_{g0}+x_{i}^{T}B\theta_{g}.$$

In the network-based multi-class classification, the log-likelihood function takes on the following form

$$\ell \ell (B,\Theta )=\sum_{i=1}^{n}(\sum_{g=1}^{G}z_{ig}(\theta_{g0}+x_{i}^{T}B\theta_{g})-\text{log}\{\sum_{g=1}^{G}\text{exp}(\theta_{g0}+x_{i}^{T}B\theta_{g})\}).$$ (9)

The network-based multi-class classification in (9) is based on the pre-estimated gene network B. Thus, we cannot effectively interpret the classification results based on the network, because any information of the status of cell line was incorporated in to the network estimation process.

To derive a gene regulatory network that is optimized for multi-class classification, we consider the following strategy that involves the simultaneous estimation of gene networks and cell line classification, following the approach presented by Park *et al*. [9],

$$\underset{B,\Theta }{\text{arg\textasciitilde{}min}}[-\sum_{i=1}^{n}(\sum_{g=1}^{G}z_{ig}(\theta_{g0}+x_{i}^{T}B\theta_{g})-\text{log}\{\sum_{g}^{G}\text{exp}(\theta_{g0}+x_{i}^{T}B\theta_{g})\})\;\;+\frac{1}{2}\sum_{j=1}^{k}\sum_{i=1}^{n}||y_{ij}-x_{i}^{T}\beta_{j}|{|}^{2}].$$ (10)

Our approach aims to estimate the gene regulatory network B in a manner that minimizes not only the errors associated with gene network estimation but also the negative log-likelihood pertaining to multi-class classification. That is, the resultant estimated network B represents the optimized gene regulatory network tailored for cell line classification. Consequently, we are able to carry out biologically meaningful interpretations for both the outcomes of gene network estimation and the multi-class classification of cell lines.

In order to address the limitations of the maximum likelihood method, we consider penalized multinormial logistic regression based on the regularization approaches. Moreover, we integrate the following insights from network biology into the statistical model to attain outcomes that are more biologically reliable results.

- Genes linked in the networks may have analogous biological functions.
- The hub genes, involved in interactions with a multitude of other genes, play key roles in governing the function and expression of multiple target genes. Disruption or dysfunction in these genes can undermine the intricate balance of gene networks, resulting in profound subsequent influences on cellular functions and disease phenotypes [15]

The knowledge of network biology can be incorporated by using the network-constrained regularization [16]. The estimated gene network from the second term in (10) can be represented by a weighted graph G=(V,E,W), where V={1,...,p} is the set of vertices corresponding to *p* genes and E∈V×V is the set of edges (i.e., pair (*i*,*j*), where i,j∈V and (i,j)∈E⇔(j,i)∈E). $W=(w_{ij}),(i,j)\in E$ is the edge weight. The normalized Laplacian matrix L for the graph is given as [16].,

$$L=l_{ij}=\left\{ \begin{array}{cc} 1-\frac{w_{ij}}{d_{i}} & \text{if }i=j\text{ and }d_{i}\neq 0,\\ -\frac{w_{ij}}{\sqrt{d_{i}d_{j}}} & \text{if }(i,j)\in E,\\ 0 & \text{otherwise} \end{array}\right.$$ (11)

where *d*_*i*_ is the degree of each gene, which is given as $d_{i}=\sum_{i\sim j}w_{ij}$.

In our method, the estimated effect of regulators on their target genes is presented within the matrix B, where each row and column of B corresponds to the index of a regulator and a target gene, respectively. We compute the weight of edges $W=w_{ij}$ based on the effect of the ${\text{i}}^{\text{th}}$ gene to ${\text{j}}^{\text{th}}$ gene (i.e., $\beta_{ij}$) and the ${\text{j}}^{\text{th}}$ gene to ${\text{i}}^{\text{th}}$ gene (i.e., $\beta_{ji}$) as follows,

$$W=w_{ij}=\frac{\left|\beta_{ij}|+|\beta_{ji}\right|}{2}.$$ (12)

We calculate the Laplacian matrix using the edge weights W, and subsequently, we integrate the estimated network into the penalized multinomial logistic regression model utilizing the Laplacian matrix L.

**Fig 1:**
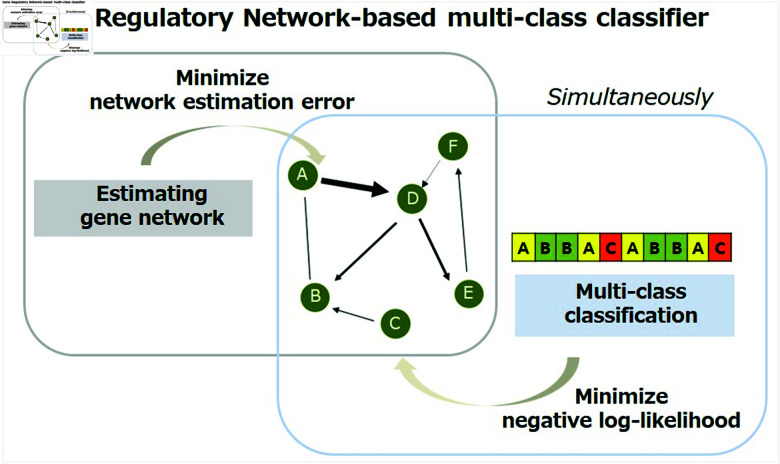
Overall framework of GRN-multiClassifier.

We then proposed the following Gene Regulatory Network based multi-class Classifier (GRN-multiClassifier),

$$\underset{B,\Theta }{\text{arg\textasciitilde{}min}}[-\sum_{i=1}^{n}(\sum_{g=1}^{G}z_{ig}(\theta_{g0}+x_{i}^{T}B\theta_{g})-\text{log}\{\sum_{g}^{G}\text{exp}(\theta_{g0}+x_{i}^{T}B\theta_{g})]\})\;\;+\frac{1}{2}\sum_{j=1}^{k}\sum_{i=1}^{n}||y_{ij}-x_{i}^{T}\beta_{j}|{|}^{2}\;\;+\lambda_{1}\sum_{j=1}^{k}||\beta_{j}||+\frac{\lambda_{2}}{2}\sum_{j=1}^{k}||\beta_{j}|{|}^{2}+\lambda_{3}\sum_{g=1}^{G}||\theta_{g}||+\frac{\lambda_{4}}{2}\sum_{g=1}^{G}\theta_{g}^{T}L^{s}\theta_{g}]$$ (13)

$$=\underset{B,\Theta }{\text{arg\textasciitilde{}min}}[-\sum_{i=1}^{n}(\sum_{g=1}^{G}z_{ig}(\theta_{g0}+x_{i}^{T}B\theta_{g})-\text{log}\{\sum_{g}^{G}\text{exp}(\theta_{g0}+x_{i}^{T}B\theta_{g})]\})\;\;+\frac{1}{2}\sum_{j=1}^{k}\sum_{i=1}^{n}||y_{ij}-x_{i}^{T}\beta_{j}|{|}^{2}\;\;+\lambda_{1}\sum_{j=1}^{k}||\beta_{j}||+\frac{\lambda_{2}}{2}\sum_{j=1}^{k}||\beta_{j}|{|}^{2}\;\;+\lambda_{3}\sum_{g=1}^{G}||\theta_{g}||+\frac{\lambda_{4}}{2}\sum_{g=1}^{G}\sum_{q=1}^{k}\sum_{j=1}^{k}(\frac{\text{sgn}(\theta_{gq})\theta_{gq}}{\sqrt{d_{q}}}-\frac{\text{sgn}(\theta_{gj})\theta_{gj}}{\sqrt{d_{j}}}{)}^{2}w_{qj}],$$

where $L_{g}^{s}=S^{T}LS$ with $S=\text{diag}(\text{sgn}({\hat{\theta }}_{g1}),...,\text{sgn}({\hat{\theta }}_{gk}))$ [17]. The proposed GRN-multiClassifier can estimate gene regulatory network that is optimized to multi-class classification. As a result, we are enabled to carry out biologically credible interpretation of the classification outcomes, grounded in the gene network. Furthermore, our method encourages similarity in gene coefficients for genes that share common edges. This is facilitated by giving considerable weight to the distinction between coefficients of genes that share a substantial number of edges. Consequently, our approach has the ability to simultaneously identify connected genes within the network. Furthermore, our approach enforces a relatively slight penalty on hub genes making it possible for these hub genes allowing hub genes to be readily recognized as key features for the classification of cancer-related statuses. In summary, our model integrates network biology knowledge, enabling robust gene network analysis and accurate multi-class classification of cell lines with a strong biological foundation. We expect that the proposed GRN-multiClassifier will be a useful tool for identifying crucial molecular interplays to characterize diseases related phenotypes. Figure 1 shows overall framework of the proposed GRN-multiClassifier.

### Implementation

The optimization of the unknown parameters $\theta_{0},\Theta$ is nonlinear and the equation does not have explicit solution. The solution, $\theta_{0},\Theta$ in this case can be obtained by using iterative algorithm. We consider Fisher’s score method to estimate the proposed GRN-multiClassifier.

By refer to the Fisher’s scoring algorithm in Appendix section, the quadratic approximation of the objective function of the GRN-multiClassifier in (13) is given as

$$\underset{B,\Theta }{\text{arg\textasciitilde{}min}}\{\frac{1}{2}\sum_{i=1}^{n}\sum_{g=1}^{G}\zeta_{ig}(k_{ig}-\theta_{g0}+x_{i}^{T}B\theta_{g}{)}^{2}+\frac{1}{2}\sum_{j=1}^{k}\sum_{i=1}^{n}||y_{ij}-\beta_{j}^{T}x_{i}|{|}^{2}\;+\lambda_{1}\sum_{j=1}^{k}||\beta_{j}||+\frac{\lambda_{2}}{2}\sum_{j=1}^{k}||\beta_{j}|{|}^{2}\;+\lambda_{3}\sum_{g=1}^{G}||\theta_{g}||+\frac{\lambda_{4}}{2}\sum_{g=1}^{G}\sum_{q=1}^{k}\sum_{j=1}^{k}(\frac{\text{sgn}(\theta_{gq})\theta_{gq}}{\sqrt{d_{q}}}-\frac{\text{sgn}(\theta_{gj})\theta_{gj}}{\sqrt{d_{j}}}{)}^{2}w_{qj}\},$$ (14)

where $k_{ig}=\theta_{g0}+x_{i}^{T}B\theta_{g}+\frac{Z_{ig}-\pi_{g}(x_{i},B)}{\pi_{g}(x_{i},B)\{1-\pi_{g}(x_{i},B)\}}$ and $\zeta_{gi}=\pi_{g}(x_{i},B)\{1-\pi_{g}(x_{i},B)\}$.

We propose the coordinate descent algorithm to optimize $\theta_{g0},\Theta$ and B. The coordinate update to optimize $\beta_{jl}$ given $\theta_{g0},\Theta$ has the following form,

$$\beta_{jl}\leftarrow \frac{S(\sum_{g=1}^{G}\theta_{gj}\sum_{i=1}^{n}x_{il}\{\zeta_{ig}(k_{ig}-k_{ig}^{\left(jl\right)})\}+\sum_{i=1}^{n}\{x_{il}(y_{ij}-y_{ij}^{\left(l\right)})\},\lambda_{1})}{\sum_{i=1}^{n}x_{il}^{2}+\sum_{g=1}^{G}\theta_{gj}^{2}\sum_{i=1}^{n}\zeta_{i}x_{il}^{2}+\lambda_{2}}$$ (15)

where


$$k_{ig}^{\left(jl\right)}=\theta_{g0}+\sum_{j=1}^{k}\sum_{r\neq l}\theta_{gj}\beta_{jr}x_{ir}+\sum_{s\neq j}\theta_{gs}\beta_{sl}x_{il}\;\text{and}\;y_{ij}^{\left(l\right)}=\sum_{r\neq l}\beta_{jr}x_{ir},$$

and S(δ,λ) is a soft thresholding operator with value

$$S(a,b)=\left\{ \begin{array}{cc} a-b & \text{if }a>0\text{ and }b<|a|,\\ a+b & \text{if }a<0\text{ and }b<|a|,\\ 0 & \text{if }b\geq |a|. \end{array}\right.$$ (16)

The sign adjusted Laplacian matrix is computed as $L_{g}^{s}=S^{T}LS$. Then, the parameters $\theta_{gj}$ are estimated by the following coordinate-wise update

$$\theta_{gj}\leftarrow \frac{S(\sum_{i=1}^{n}\sum_{g=1}^{G}\zeta_{ig}(k_{ig}-k_{i}^{\left(j\right)})\beta_{j}^{T}x_{i}-\lambda_{4}\sum_{c\neq j}\theta_{gc}l_{cj}^{s},\lambda_{3})}{\sum_{i=1}^{n}\zeta_{ig}(\beta_{j}^{T}x_{i}{)}^{2}+\lambda_{4}l_{jj}^{s}}$$ (17)

where $l_{cj}^{s}$ is ${\text{j}}^{\text{th}}$ element in *c* row of $L^{s}$ and $k_{ig}^{\left(j\right)}=\theta_{g0}+\sum_{c\neq j}\theta_{gc}\beta_{c}^{T}x_{i}$.

Given B and Θ, coordinate $\theta_{g0}$ is given as

$${\hat{\theta }}_{g0}=\frac{1}{\sum_{i=1}^{n}\zeta_{gi}}\sum_{i=1}^{n}\zeta_{gi}(k_{ig}-\sum_{j=1}^{k}\theta_{gj}\sum_{l=1}^{p}\beta_{jl}x_{il}).$$ (18)

The parameters B,Θ and $\theta_{g0}$ are cyclically updated until convergence.

### Regularization parameters selection

The results of the proposed GRN-multiClassifier heavily rely on the regularization parameters $\lambda_{1},\lambda_{2},\lambda_{3}$ and $\lambda_{4}$. The $\lambda_{1},\lambda_{2}>0$ and $\lambda_{3},\lambda_{4}>0$ are regularization parameters of $\beta_{j}$ and $\theta_{g}$, respectively. When $\lambda_{2}=0$ and $\lambda_{1}>0$ ($\lambda_{4}=0$, $\lambda_{3}>0$), the penalty of $\beta_{j}$ ($\theta_{g}$) is reduced to the lasso. On the other hand, for $\lambda_{1}$ and $\lambda_{2}>0$ ($\lambda_{3}$ and $\lambda_{4}>0$) retains the good properties of both sparsity and smoothness for edge estimation in a network, i.e., ${\hat{beta}}_{j}$, (predictor variable estimation in classification model, i.e., ${\hat{\theta }}_{g}$).

We consider the following Bayesian Information Criterion (BIC) to select the regularization parameters,

$$BIC=-2\sum_{i\in D_{vl}}(\sum_{g=1}^{G}z_{ig}({\hat{\theta }}_{g0}^{tr}+x_{i}^{T}{\hat{B}}^{tr}{\hat{\theta }}_{g}^{tr})-\text{log}\{\sum_{g=1}^{G}\text{exp}({\hat{\theta }}_{g0}^{tr}+x_{i}^{T}{\hat{B}}^{tr}{\hat{\theta }}_{g}^{tr})\})+\text{log}(n_{vl})df,$$ (19)

where $D_{vl}$ the set of indexes of the validation dataset, $n_{vl}$ is a number of observations of the validation dataset, ${\hat{\theta }}_{g0}^{tr},{\hat{B}}^{tr},{\hat{\theta }}_{g}^{tr}$ are estimated parameters based on training dataset, and *df* is the degree of freedom of the GRN-multiClassifier. We use the number of nonzero elements in $\theta_{g},g=1,...,G$ as an estimate of the degrees of freedom in line with [9, 18, 19]. We apply our method to acute leukemia subtypes classification based on dataset from the R package *golubEsets* (https://jokergoo.github.io/cola_examples/Golub_leukemia/).

## Monte Carlo simulations

We demonstrated the effectiveness of the GRN-multiClassifier through the utilization of Monte Carlo simulations. We assumed that each transcription factor gene (TF) regulates a set of 10 genes, and the expression levels of these transcription factor genes were generated from *N*(0,1). The expression levels of each of the regulated genes ($y_{j}$, j=1,...10) by the TF ($x_{t}$) were generated in accordance with the expression level of the TFs as follows,


$$y_{ij}=x_{it}\beta_{jt}+\epsilon_{ij}^{y},\;i=1,...,n,\;j=1,...,10.$$

where $\epsilon_{ij}^{y}\sim N(0,1)$.

The response variable $z_{i}=(z_{i1},...,z_{iG})$ is generated by taking into account the regulatory effect of genes, that is, both the gene expression levels denoted as X and the effect of regulators on their targets represented by the matrix $B=(\beta_{1},...,\beta_{k})$.

We conducted simulations with 50 datasets generated from the following true model,


$$z_{i}\sim \text{Multinomial}(\pi_{1}(x_{i},B),...,\pi_{G}(x_{i},B)),\;\text{log}\frac{\pi_{g}(x_{i},B)}{\pi_{G}(x_{i},B)}=\theta_{g0}+x_{i}^{T}B\theta_{g}$$

We set *G* = 3 and assume that the data were simulated based on the scenarios established as benchmarks in prior studies [9, 16].

**Scenario 1:**


$$\beta_{jt}=0.7,\;j=1,...,10,\;t=1,...,T$$

$$\theta_{g}=(1,\underset{10}{\underbrace{\frac{1}{\sqrt{5}},...,\frac{1}{\sqrt{5}}}},-1,\underset{10}{\underbrace{\frac{-1}{\sqrt{20}},...,\frac{-1}{\sqrt{20}}}},0.8,\underset{10}{\underbrace{\frac{0.8}{\sqrt{5}},...,\frac{0.8}{\sqrt{5}}}},-0.8,\underset{10}{\underbrace{\frac{-0.8}{\sqrt{20}},...,\frac{-0.8}{\sqrt{20}}}},0,...,0),$$

g=1,2,3

**Scenario 2:**


$$\beta_{jt}=0.7,\;j=1,...,10,\;t=1,...,T$$

$$\theta_{g}=(1,\underset{10}{\underbrace{\frac{1}{\sqrt{10}},...,\frac{1}{\sqrt{10}}}},-1,\underset{10}{\underbrace{\frac{-1}{\sqrt{10}},...,\frac{-1}{\sqrt{10}}}},0.8,\underset{10}{\underbrace{\frac{0.8}{\sqrt{10}},...,\frac{0.8}{\sqrt{10}}}},-0.8,\underset{10}{\underbrace{\frac{-0.8}{\sqrt{10}},...,\frac{-0.8}{\sqrt{10}}}},0,...,0),$$

g=1,2,3

**Scenario 3:**


$$\beta_{jt}=0.9,\;j=1,...,5,\;t=1,...,T$$

$$\beta_{jt}=0.7,\;j=6,...,10,\;t=1,...,T$$

$$\theta_{g}=(1,\underset{10}{\underbrace{\frac{1}{\sqrt{5}},...,\frac{1}{\sqrt{5}}}},-1,\underset{10}{\underbrace{\frac{-1}{\sqrt{20}},...,\frac{-1}{\sqrt{20}}}},0.8,\underset{10}{\underbrace{\frac{0.8}{\sqrt{5}},...,\frac{0.8}{\sqrt{5}}}},-0.8,\underset{10}{\underbrace{\frac{-0.8}{\sqrt{20}},...,\frac{-0.8}{\sqrt{20}}}},0,...,0),$$

g=1,2,3

**Scenario 4:**


$$\beta_{jt}=0.9,\;j=1,...,5,\;t=1,...,T$$

$$\beta_{jt}=0.7,\;j=6,...,10,\;t=1,...,T$$

$$\theta_{g}=(1,\underset{10}{\underbrace{\frac{1}{\sqrt{5}},...,\frac{1}{\sqrt{5}}}},-1,\underset{10}{\underbrace{\frac{-1}{\sqrt{20}},...,\frac{-1}{\sqrt{20}}}},0.8,\underset{10}{\underbrace{\frac{0.8}{\sqrt{5}},...,\frac{0.8}{\sqrt{5}}}},-0.8,\underset{10}{\underbrace{\frac{-0.8}{\sqrt{20}},...,\frac{-0.8}{\sqrt{20}}}},0,...,0),$$

g=1,2,3

We consider the number of observations *n* = 150 consisting of training, validation and test dataset with 80%, 10%, and 10% of 150 observations, respectively. In each scenario, we consider number of TFs (i.e, *T*) as 10 and 20. We assess the performance of the proposed Gene Regulatory Network based multi-class Classifier (GNmC) by comparing it with a multi-class classification model grounded in a pre-estimated network (prNW). The gene network for the prNW model is obtained through the utilization of the lasso. We further conduct a comparison among classification methods based on not the network, but the expression levels of genes X, where lasso (LA), elastic net (ELA), kernelized support vector machines (KSVM) and random forest (RF) are used for classification of cell lines. That is, the GNmC and prNW are the network-based classification, while LA, ELA, KSVM and RF are the expression-based classification.

We also consider the scenarios for networks with the regulators comprising both activators and inhibitors, as described below:

**Scenarios 1 and 2:**


$$\beta_{jt}=0.7,\;j=1,...,5,\;t=1,...,T$$

$$\beta_{jt}=-0.7,\;j=6,...,10,\;t=1,...,T$$

**Scenario 3 and 4:**


$$\beta_{jt}=0.9,\;j=1,...,5,\;t=1,...,T$$

$$\beta_{jt}=-0.7,\;j=6,...,10,\;t=1,...,T$$

In the situation for networks consisting of activators and inhibitors, the Θ is given in same as the scenarios for networks consisting of activators only.

We compare the multi-class classification accuracy in Table 1. As indicated in Table 1, the network-based methods (i.e., GRN-multiClassifier and prNW) exhibit exceptional performance in terms of multi-class classification accuracy compared with expression levels based approaches (i.e., ELA, LA, KSVM and RF). Furthermore, it can be seen that the proposed GRN-multiClassifier provides the most effective multi-class classification results in overall. We also evaluate our method for the feature selection accuracy, encompassing true positive rates, true negative rates, and their averages, for both Θ in the multinomial logistic regression model and B in the network estimation. [Simul_FeatureS]Table 2 shows the feature selection results. From the perspective of feature selection accuracy for Θ, the network-based methods demonstrate effective outcomes in comparison to approaches based on expression levels. Our approach demonstrates remarkable outcomes in terms of feature selection, encompassing not only multinomial logistic regression but also the selection of edges in gene regulatory network estimation. The results clearly indicate that the proposed GRN-multiClassifier delivers exceptional performance across gene network estimation, feature selection, and multi-class classification of cell lines.

**Table 1:** Multi-class classification accuracy, where GNmC: GRN-multiClassifier, prNW: multi-class classification model grounded in a pre-estimated network, Classification results based on expression levels by using LA: lasso, ELA: elastic net, KSVM: kernelized support vector machine, RF: random forest.

	No.TF	Scenarios	Methods					
			GNmC	prNW	ELA	LA	KSVM	RF
Activators	10	1	**0.916**	0.907	0.844	0.855	0.764	0.744
		2	**0.915**	0.909	0.849	0.864	0.784	0.731
		3	**0.915**	0.905	0.831	0.835	0.775	0.751
		4	**0.913**	0.905	0.821	0.849	0.731	0.701
	20	1	**0.855**	0.852	0.817	0.828	0.872	0.836
		2	**0.888**	0.868	0.852	0.856	0.695	0.692
		3	**0.888**	0.875	0.853	0.863	0.709	0.703
		4	**0.876**	**0.876**	0.820	0.824	0.699	0.685
Activators & Inhibitors	10	1	**0.928**	0.904	0.844	0.856	0.769	0.737
		2	**0.921**	0.904	0.844	0.861	0.791	0.713
		3	**0.921**	0.899	0.827	0.844	0.744	0.704
		4	0.921	**0.936**	0.833	0.843	0.765	0.724
	20	1	**0.887**	0.873	0.831	0.839	0.739	0.736
		2	**0.885**	0.851	0.823	0.841	0.731	0.715
		3	**0.885**	0.871	0.833	0.839	0.680	0.693
		4	**0.883**	0.876	0.859	0.863	0.657	0.664

**Table 2:** Simulation results of networks selection in multinormial logistic regression and edge selection in network estimation, where GNmC: GRN-multiClassifier, prNW: multi-class classification model grounded in a pre-estimated network, Classification results based on expression levels by using LA: lasso, ELA: elastic net, KSVM: kernelized support vector machine, RF: random forest.

	No.TF	Scenario	Network selection $\hat{\Theta }$												Feature selection of Edges $\hat{B}$					
			TPR				TNR				Average				TPR		TNR		Average	
			GNmC	prNW	ELA	LA	GNmC	prNW	ELA	LA	GNmC	prNW	ELA	LA	GNmC	prNW	GNmC	prNW	GNmC	prNW
Activators	10	1	0.91	0.86	0.27	0.22	0.66	0.66	0.89	0.92	**0.78**	0.76	0.58	0.57	1.00	1.00	0.99	0.96	**1.00**	0.98
		2	0.92	0.91	0.29	0.23	0.67	0.63	0.87	0.91	**0.80**	0.77	0.58	0.57	1.00	1.00	0.99	0.96	**1.00**	0.98
		3	0.92	0.85	0.27	0.22	0.67	0.66	0.88	0.90	**0.80**	0.75	0.57	0.56	1.00	1.00	0.99	0.96	**1.00**	0.98
		4	0.84	0.87	0.28	0.23	0.71	0.67	0.88	0.91	**0.78**	0.77	0.58	0.57	0.99	1.00	1.00	0.96	**0.99**	0.98
	20	1	0.90	0.85	0.22	0.19	0.74	0.75	0.94	0.95	**0.82**	0.80	0.58	0.57	1.00	1.00	1.00	0.98	**1.00**	0.99
		2	0.91	0.90	0.25	0.20	0.74	0.72	0.93	0.95	**0.83**	0.81	0.59	0.57	1.00	1.00	1.00	0.98	**1.00**	0.99
		3	0.91	0.85	0.24	0.19	0.74	0.75	0.92	0.95	**0.83**	0.80	0.58	0.57	1.00	1.00	1.00	0.98	**1.00**	0.99
		4	0.85	0.87	0.21	0.19	0.78	0.76	0.94	0.95	**0.81**	**0.81**	0.58	0.57	0.99	1.00	1.00	0.98	**0.99**	**0.99**
Activators & Inhibitors	10	1	0.91	0.91	0.31	0.23	0.66	0.63	0.85	0.92	**0.79**	0.77	0.58	0.57	1.00	1.00	0.99	0.96	**1.00**	0.98
		2	0.91	0.92	0.33	0.23	0.65	0.63	0.84	0.91	**0.78**	0.77	0.59	0.57	1.00	1.00	0.99	0.96	**1.00**	0.98
		3	0.91	0.86	0.31	0.23	0.65	0.67	0.85	0.91	**0.78**	0.77	0.58	0.57	1.00	1.00	0.99	0.96	**1.00**	0.98
		4	0.84	0.86	0.26	0.22	0.72	0.66	0.89	0.91	**0.78**	0.76	0.57	0.57	0.99	1.00	1.00	0.96	**0.99**	0.98
	20	1	0.91	0.91	0.25	0.20	0.74	0.73	0.93	0.95	**0.83**	0.82	0.59	0.58	1.00	1.00	1.00	0.98	**1.00**	0.99
		2	0.90	0.90	0.24	0.20	0.73	0.73	0.93	0.95	**0.82**	0.82	0.59	0.57	1.00	1.00	1.00	0.98	**1.00**	0.99
		3	0.90	0.86	0.24	0.20	0.73	0.75	0.93	0.95	**0.82**	0.81	0.58	0.57	1.00	1.00	1.00	0.98	**1.00**	0.99
		4	0.85	0.87	0.23	0.20	0.78	0.75	0.93	0.95	**0.82**	0.81	0.58	0.57	0.99	1.00	1.00	0.98	**0.99**	**0.99**

## Gene regulatory network based acute leukemia subtypes classification

Acute leukemias are malignancies that originate from either the lymphoid or myeloid cell line and are characterized by rapid and uncontrolled proliferation of immature blood cells. AML is a cancerous condition that affects myeloid cells, responsible for generating certain white blood cells, whereas Acute Lymphocytic Leukemia (ALL) primarily impacts lymphocytes, a critical component of the immune system’s white blood cells. AML is a highly aggressive cancer that tends to progress rapidly and can be particularly deadly and thus uncovering the molecular interplays that play key role in AML-related mechanism is a crucial issue. We apply the proposed GRN-multiClassifier to estimate a gene network that is optimized to provide insights into the characteristics of acute leukemia cell lines. We consider the well-known acute leukemia gene expression dataset [10], which has been explored in various fields of research on multiclass cancer analysis [21, 22]. The data set consist of 72 cell lines for 7129 genes that are available in the R package *golubEsets* (https://jokergoo.github.io/cola_examples/Golub_leukemia/).

Diagnosing acute leukemia can be framed as a tri-classification problem, i.e., B-cell acute lymphoblastic leukemia (BALL), T-cell acute lymphoblastic leukemia (TALL), and AML [23, 24], where the dataset consisting of 38 BALL cell lines, 9 TALL cell lines, and 25 AML cell lines. In this study, we consider the classification of cell lines into the tree types of acute leukemia (*g* = 1: AML; *g* = 2:BALL; *g* = 3:TALL) based on the gene regulatory networks.

It is known that extremely high dimensional data situation can cause poor performance for machine learning model [20] and lead to difficulty in visualization of the results. Thus, we peroform multi-class classification based on 50, 100, 200, 300, 400, and 500 genes with the highest variance in 72 cell lines. The training and test dataset consist of 57 (80%) and 15 (20%) cell lines, respectively.

**Fig 2:**
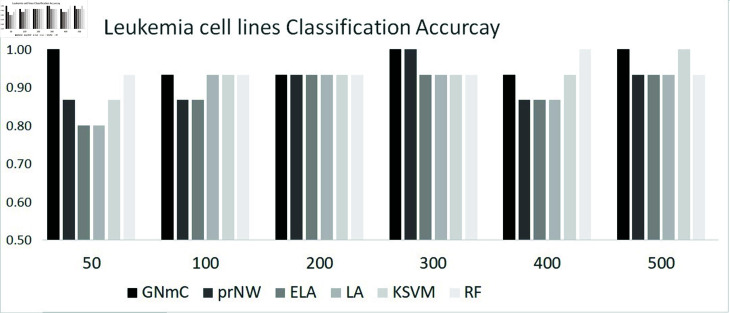
Leukemia cell line classification results, where GNmC: GRN-multiClassifier, prNW: multi-class classification model grounded in a pre-estimated network, Classification results based on expression levels by using LA: lasso, ELA: elastic net, KSVM: kernelized support vector machine, RF: random forest. The AML cell lines classification is performed by 50, 100, 200, 300, 400, 500 genes having largest variances of expression levels.

Figure 2 shows the classification results. The proposed GRN-multiClassifier shows effective results in the classification of acute leukemia cell lines in overall, even though the random forest show better classification accuracy than our method.

Our strategy shows the effective classification results (i.e., 100% accuracy) based on the classifiers with 100, 300, 500 genes. Other methods (i.e., prNW, KSVM and RF) also show the effective results (i.e., 100% accuracy) based on 300, 500, 400 genes, respectively. It implies that increasing number of features cannot always improve classification accuracies. The classification utilizing the gene network comprising 50 genes achieves a perfect accuracy of 100%, where the large number of genes do not guarantee improvement of accuracy. Thus, we consider that the gene network with 50 genes is enough to explain status of cell lines, because the network achieves the perfect classification result. We proceed to interpret the outcomes of acute leukemia cell line classification based on this gene network of 50 genes. The optimized gene networks (i.e., edges) with 50, 100, 300, 500 genes estimated by our method are given in Supplementary file.

We identify genes that correspond to the largest absolute values of $\theta_{g}$, with g=1,2,3, as crucial markers, i.e., we extract the top 5 genes for each category (g=1,2,3) based on their largest absolute $\theta_{g}$ values. Table 3 shows the crucial markers and their leukemia related evidences. As shown in Table 3, 11 identified markers have been uncovered in previous studies and only a marker has no evidences.

**Table 3:** The identified markers for Leukemia cell line classification and their evidences related AML markers uncovered in previous studies.

Accession ID	*g*	Gene Name	Reference related to Leukemia
M27783_s_at	1	ELANE/ELA2	[25–27]
M20203_s_at	1		
Y00787_s_at	1	CXCL8	[28–30]
M27891_at	1	CST3	[31–35]
M19507_at	1	MPO	[36–41]
X00274_at	2	HLA-DRA	[42–44]
M13560_s_at	2	CD74	[40, 45]
X82240_rna1_at	2	TCL1A	[46, 47]
M26602_at	2	DEFA1	[48, 49]
L19779_at	2	H2AC19	-
X00437_s_at	3	TRBV19/TCRB	[50]
AFFX-HSAC07/X00351_M_at	3	ACTB	[51, 52]
AFFX-HSAC07/X00351_5_at	3		
X00351_f_at	3		
D49824_s_at	3	HLA-B	[53–55]

- *ELANE*
- In the study conducted by Yanli *et al*. [25], it was observed that heightened expression of ELANE correlated with comparatively shorter survival durations among leukemia patients. In light of this, the authors proposed that ELANE serves as an oncogene driving leukemia development. Patients harboring the ELANE mutation may experience the onset of myelodysplastic syndrome (MDS), AML, or, in rarer cases, acute lymphoblastic leukemia (ALL) [26]. Although it is vital to keep track of patients with ELANE-germline mutations, the initial leukemogenesis process in ELANE-neutropenia patients is characterized by the presence of CSF3R mutations, potentially induced by GCSF therapy and is adequate to cause myelodysplasia and acute leukemia [26].
- *CXCL8*
- Research findings indicate that the expression level of CXCL8 is positively correlated with recurrence probability in AML and CXCL8 plays significant role in promoting AML cell growth through the activation of the ERK1/2 signal pathway [28]. The CXCL8 derived from mesenchymal stromal cells promotes the survival and proliferation of AML cells by activating the PI3K/AKT pathway [29]. Significant upregulation of CCL3, CCL4, CXCL8, and IL-17A messenger RNA (mRNA) expression levels was observed in the adult T-cell leukemia/lymphoma (ATLL) groups, as reported by Soltani *et al*. [30]. The significant association between CXCL8 and ATLL was evident, and the upregulation of CXCL8 was associated with an increased odds ratio of ATLL.
- *CST3*
- CST3 has been identified as a biologically relevant gene in the context of leukemia [31], and it has also been noted as one of the up-regulated genes in patient/donor pairs with AML [32]. Elevated expressions of CSTA, CSTB, CST3, and CST7 were found to be correlated with higher percentages of monocytes and neutrophils in the peripheral blood of patients with AML, as reported by Yuita *et al*. [34]. CST3 was selected in the Top 10 ranked informative genes from the leukaemia dataset [34]. CST3 and MPO were experimentally demonstrated to exhibit a correlation with acute lymphoblastic leukemia (ALL) or AML [35]. In the study by Wang *et al*. [35], it was shown that the gene groups CST3, MPO, and IGL are strongly associated with the development of leukemia.
- *MPO*
- Itonaga *et al*. [36] revealed that MPO is linked to myeloid lineage commitment and is indicative of a favorable prognosis in patients with AML. The presence of MPO serves as an informative marker for distinguishing a distinctive and crucial DNA methylation profile in CD34-positive AML cells. Patients with B-ALL-isoMPO exhibit a greater risk of relapse than those with B-ALL [38]. MPO plays a crucial role in determining the susceptibility of leukemia cells to parthenolide-induced apoptosis [39]. Parthenolide holds promise as a potential therapy targeting leukemic stem cells, particularly for AML cases characterized by elevated levels of MPO expression. Expression of MPO was detected in a majority of AML [40]. MPO gene expression has the potential to function as an additional marker for distinguishing various types of acute leukemias. Moreover, it could aid in the identification of leukemic cells arrested during the initial stages of the myeloid differentiation pathway. The presence of elevated MPO expression, indicative of a more differentiated cellular profile, has been associated with positive clinical outcomes in AML, as outlined by Kumari *et al*. [41].
- *HLA-DRA*
- In specific human leukemia cases, the expression of HLA-DR antigens is indicative of cellular differentiation, as discussed in the work by Tobe *et al*. [42]. Dorak *et al*. [43] showed a highly significant association of a homozygous HLA-DR genotype in childhood ALL with a strong gender effect. Multiple sclerosis risk markers in HLA-DRA, HLA-C, and IFNG genes are associated with sex-specific childhood leukemia risk [44].
- *CD74*
- The expression of CD74 was detected in various AML cell lines and patient samples that exhibit sensitivity to cytotoxicity induced by the anti-CD74 treatment (milatuzumab), as demonstrated by Le *et al*. [45]. It was also demonstrated that CD74 is expressed more frequently and at higher levels on AML blasts compared to lymphocytes. The ease of targeting the phenotype and the presence of the anti-CD74 antibody milatuzumab suggest that further investigate into the role of CD74 in AML biology [40].
- *TCL1A*
- Aggarwal *et al*. [46] demonstrated the prognostic relevance of TCL1A expression in patients with chronic lymphocytic leukemia and mantle cell lymphoma. Expression and/or rearrangement of TCL1A is a useful marker to diagnosing T-cell prolymphocytic leukemia [47]. Upregulation of PAX5, CD72, CSRP2, LOC100130458, TCL1A and EBF1 genes is associated with patients diagnosed with ALL patients in a phenotype-related signature.
- *DEFA1*
- Overexpression of FLT3 and DEFA1 genes retained independent prognostic significance for B-ALL outcome [48]. The overexpressions of DEFA1-3 may be associated with an increase in malignancy during relapsed leukemia and could potentially serve as predictive markers for therapy resistance during relapse, as suggested by Te *et al*. [49]. Te Kronnie *et al*. [49] illustrated that relatively high expression levels of DEFA1-3 are linked to an unfavorable response to chemotherapy, which in turn leads to early relapse in leukemia patients. The overexpression of DEFA1-3 was found to be associated with elevated expression of both MPO and ELA2, where these latter genes are preferentially transcribed within the most immature granulocytes. There was a suggestion that the simultaneous overexpression of DEFA1-3, MPO, and ELA2 might contribute to a specific granulopoiesis signature.
- *TRBV19/TCRB*
- The presence of TCRB gene rearrangements was detected in 35% of precursor-B-ALL patients, encompassing both children and adult [50].
- *ACTB*
- ACTB was identified as a member of set for use as control/reference for the analysis of gene expression in peripheral blood and bone marrow samples from patients with acute leukemias [51]. The genes CLUS, CERU, APOE, APOA4, APOA1, GELS, S10A9, AMBP, ACTB, CATA, and AFAM have been identified as important factors in leukemia prognosis, with the potential to act as unique biomarkers for gauging the aggressiveness of leukemia or as suppressor proteins specifically in cases of high-risk acute lymphoblastic leukemia (HR-ALL), as suggested by the study referenced as [52].
- *HLA-B*
- Fernandez *et al*.[53] uncovered the contradictory effects of the HLA-B*40 allele in terms of genetic susceptibility to develop ALL or AML. The somatic mutation identified in the HLA-B gene of leukemic cells was responsible for the observed variations in typing and sequencing alterations in the peripheral blood sample, as stated in the study cited as [54]. Vikash *et al*. [55] provided the evidence of the HLA-B allele is associated with leukemia in the North Indian population.

It can be seen through literature that the crucial genes identified for the classification of acute leukemia cell lines have strong evidences as acute leukemia markers.

**Fig 3:**
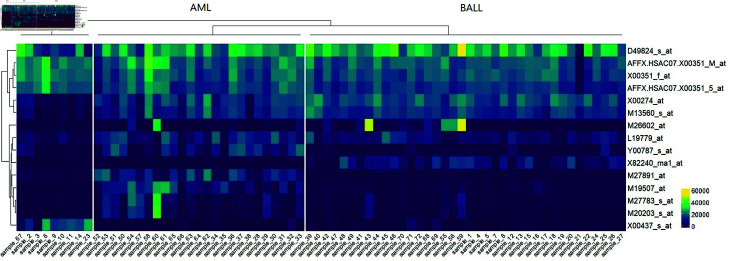
Heatmap of the identified leukemia markers in the B-cell acute lymphoblastic leukemia, T-cell acute lymphoblastic leukemia, and acute myeloid leukemia cell lines.

Figure 3 shows the heatmap of the identified markers in the BALL, TALL, and AML cell lines. The markers show different pattern in the BALL, TALL, and AML cell lines. X00274_at, M13560_s_at, L19779_at show relatively high expression levels in AML and BALL cell lines than TALL cell lines. Y00787_s_at, M27891_at, M19507_at, M27783_s_at, M20203_s_at are up-regulated in AML cell lines, while X00437_s_at is up-regulated in TALL cell lines. D49824_s_at, AFFX.HSAC07.X0035_M_at, X00351_f_at and AFFX.HSAC07.X00351_5_at show high expression levels in all acute leukemia cell line. This suggests that the ACTB gene (Accession ID: AFFX.HSAC07.X00351_M_at, X00351_f_at, and AFF X.HSAC07.X00351_5_at) holds the potential to be regarded as a critical marker for acute leukemia. Furthermore, the outcomes imply that inhibiting ACTB could potentially offer valuable insights into comprehending the mechanisms underlying acute leukemia.

**Fig 4:**
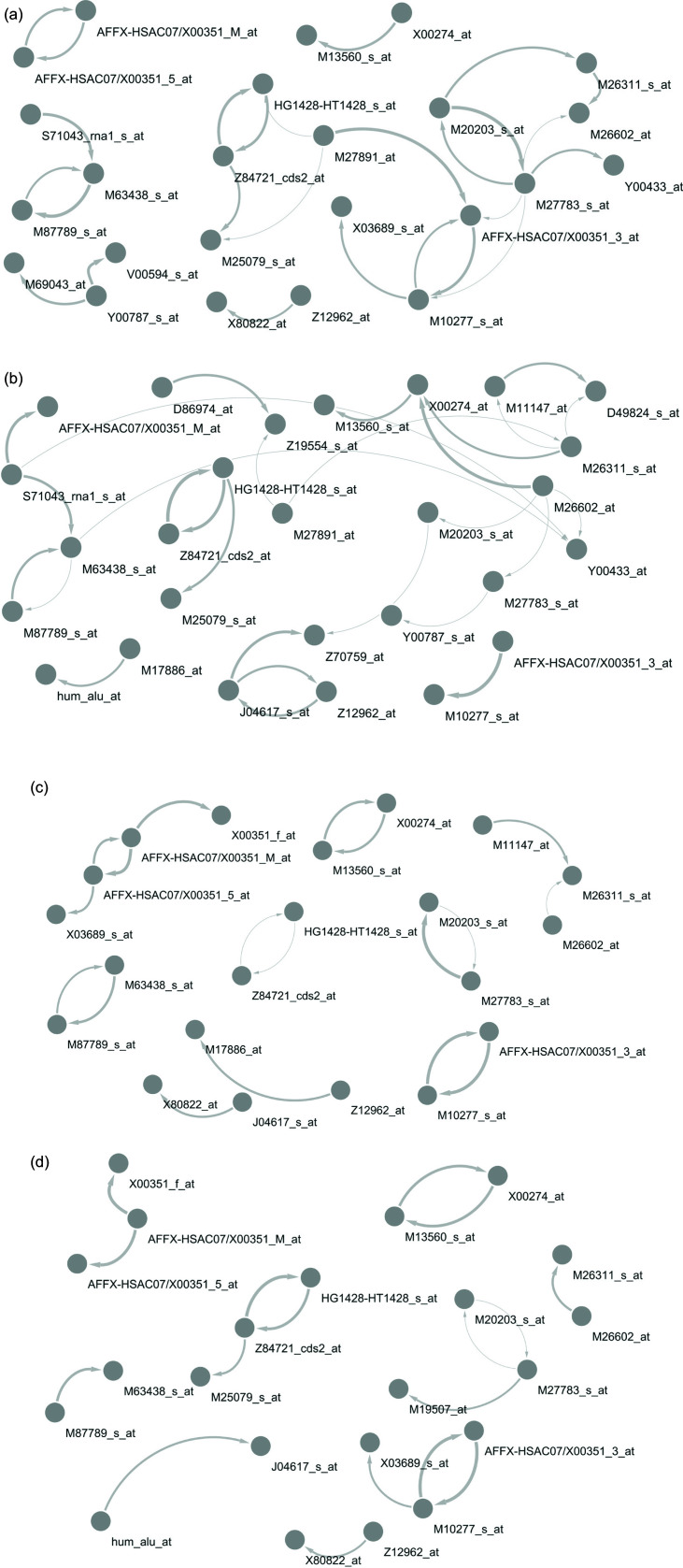
Molecular interplays of the identified acute leukemia markers in B-cell acute lymphoblastic leukemia (BALL), T-cell acute lymphoblastic leukemia (TALL), acute myeloid leukemia cell lines (AML) and the estimated network without consideration of classification (Ordinary network).

The proposed GRN-multiClassifier incorporates the *L*_1_ penalty of θ into multinomial logistic regression, and thus provides sparse estimation result for θ. It implies that we can identify crucial markers for the classification of acute leukemia cell lines. We present the networks of the identified crucial markers, i.e., the networks consist of the selected target genes ($\theta_{gj}\neq 0$) and their regulator genes. We estimate gene networks for AML, BALL, and TALL cell lines separately using the genes within the extracted network. These networks are constructed using the specific cell lines corresponding to AML, BALL, and TALL. To effectively visualize, we extract edges with an absolute value exceeding 0.5. Figure 4 shows the crucial molecular interactions for acute leukemia cell line classification in AML, BALL, TALL cell lines and the estimated gene network without consideration of classification (ordinary network), where the ordinary network describes not optimized molecular interplays for leukemia cell line classification but the original network without consideration of the classification. As shown in Figure 4, the networks within AML cell lines display sparse molecular interactions, a dense gene network is observed in TALL cell lines, where numbers of edges are 18, 26, and 28 in the networks of AML, BALL and TALL cell lines, respectively. The ordinary network shows the relatively sparse network, i.e., the network consists of 18 edges. The AML and BALL cell lines exhibit analogous molecular interactions, specifically sharing 11 common edges in their respective networks. Conversely, the TALL cell lines feature distinct gene networks when compared to the networks of AML and BALL cell lines. In detail, the networks of AML and TALL cell lines share 6 common edges, while the networks of BALL and TALL cell lines share 7 common edges. The ordinary network relatively larger number of common edges with the networks of AML and BALL cell lines, while the network of TALL cell lines shows differentially regulated gene network with those of the ordinary network. The results imply that the ordinary network describes the general molecular interplays for the network of AML, BALL and TALL cell lines, where 12 edges among the 18 edges of the ordinary network are existed in the networks of AML and BALL cell lines. On the other hand, the optimized gene networks for leukemia cell line classification show relatively distinguishing molecular interplays for each cell lines. Especially, the T-cell acute lymphoblastic leukemia may possess distinct molecular characteristics that set it apart from AML and BALL.

For the genes in the optimized networks for the leukemia cell line classification (i.e., genes in the networks of AML, BALL and TALL cell lines in Figure 4), we perform differentially expression genes analysis. Table 4 show the results of analysis of variance (ANOVA). Among the 33 genes in the optimized networks, 17 genes (51.2%) show significant difference (Pvalue<0.05) of expression levels in AML, BALL and TALL cell lines, while only 23.2% of the non-selected genes show significantly different expression levels. The results imply that our strategy for the optimized gene networks analysis for cell line classification can identify crucial genes to characterize status of cell lines in the viewpoint of expression levels. It can be considered that the molecular interplays of the remain 16 genes corresponding Pvalue larger than 0.05 are crucial features for classification, even though the expression levels of the genes are not differently expressed between the AML, BALL and TALL cell lines. Thus, the genes can be considered as novel founding (i.e., candidate markers) for leukemia cell line classification,which cannot be revealed by widely used single gene-based analysis (e.g., differentially expressed gene analysis).

**Table 4:** Differentially expressed genes analysis

Genes	F-stat	Pvalue	Genes	F-stat	Pvalue
Y00433_at	42.80	0.00	X00351_f_at	2.08	0.15
M27891_at	38.97	0.00	D49824_s_at	1.30	0.26
M11147_at	28.12	0.00	J04617_s_at	1.19	0.28
Y00787_s_at	23.69	0.00	Z12962_at	0.91	0.34
M69043_at	14.70	0.00	X80822_at	0.79	0.38
M27783_s_at	10.17	0.00	Z84721_cds2_at	0.77	0.38
M63438_s_at	9.84	0.00	M10277_s_at	0.74	0.39
M26311_s_at	9.62	0.00	AFFX-HSAC07/X00351_3_at	0.69	0.41
M87789_s_at	9.41	0.00	AFFX-HSAC07/X00351_5_at	0.62	0.43
S71043_rna1_s_at	9.19	0.00	Z70759_at	0.55	0.46
M20203_s_at	8.92	0.00	HG1428-HT1428_s_at	0.41	0.52
M13560_s_at	7.33	0.01	M25079_s_at	0.15	0.70
V00594_s_at	6.82	0.01	AFFX-HSAC07/X00351_M_at	0.08	0.77
X00274_at	4.79	0.03	D86974_at	0.05	0.83
Z19554_s_at	4.59	0.04	M26602_at	0.00	0.97
X03689_s_at	4.46	0.04			
M17886_at	4.30	0.04			
hum_alu_at	2.54	0.12			
Mean of the selected 33 genes	13.35	0.02	Significant genes: 51.1% (♯ Pvalue<0.05)		
Mean of the non selected 7096 genes	3.09	0.36	Significant genes: 23.2% (♯ Pvalue<0.05)		
Mean of total 7129 genes	3.11	0.36	Significant genes: 23.3% (♯ Pvalue<0.05)		

**Fig 5:**
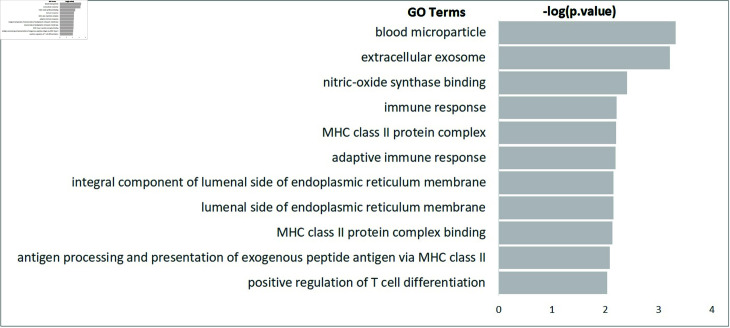
GO term pathway analysis of the genes in the identified crucial common edges for three type of acute leukemia cell lines classification: enriched GO term with p.value<0.01 and -log(*p*.*value*).

We extract the common edges from the three gene networks for AML, BALL and TALL cell lines in Table 5. To comprehend the biological processes implicated in the shared gene regulatory system of acute leukemia cell lines, we conducted a Gene Ontology (GO) term pathway analysis of the genes present in the common edges. Figure 5 illustrates the enriched pathways, with the p-value represented as -log(p-value). As shown in Figure 5, “blood microparticle" is the most enriched pathway of the genes in common edges for AML, BALL and TALL cell lines. The immune response related pathways (“immune response" and “adaptive immune response") are also enriched for the common markers of acute leukemia cell lines. Furthermore, lumenal side of endoplasmic reticulum membrane-related pathways (“integral component of lumenal side of endoplasmic reticulum membrane" and “lumenal side of endoplasmic reticulum membrane") are also identified as enriched pathways for the common markers.

**Table 5:** Identified common edges in gene networks for AML, BALL and TALL cell lines shown in Figure 3. The common edges are existing in three gene networks estimated by BALL, TALL, AML cell lines.

Regulators		Targets	
Accession ID	Gene name	Accession ID	Gene name
Z84721_cds2_at	HBA1	HG1428-HT1428_s_at	HBB
HG1428-HT1428_s_at	HBB	Z84721_cds2_at	HBA1
M63438_s_at	IGKV1-5	M87789_s_at	IGHV4-31
M87789_s_at	IGHV4-31	M63438_s_at	IGKV1-5
X00274_at	HLA-DRA	M13560_s_at	CD74
AFFX-HSAC07/X00351_3_at	ACTB	M10277_s_at	ACTB

Table 6 show the leukemia related evidences for the identified GO terms of the crucial common edges. The uncovered biological pathways for common genes in the networks were identified as crucial biological functions to AML mechanism by the Gene Ontology (GO) term analysis of differentially expression genes analysis.

**Table 6:** Evidences for the Leukemia of the identified biological pathways.

GO terms	Evidences
Blood microparticle	[56–59]
Nitric-oxide synthase binding	[60]
Extracellular exosome	-
Immune response	[61–64]
MHC class II protein complex	-
Adaptive immune response	[65]
Integral component of lumenal side of endoplasmic reticulum membrane	-
Iumenal side of endoplasmic reticulum membrane	[66]
MHC class II protein complex binding	-
Antigen processing and presentation of exogenous peptide antigen via MHC class II	[67]
Positive regulation of T cell differentiation	-

- *Blood microparticle*
- The GO term *Blood microparticle* was revealed as an enriched pathway related differentially expressed genes (DEGs) related leukemia cells as follow. A comparison of differential mRNAs and miRNAs between the pAML and control groups demonstrated that pediatric AML DEGs are predominantly enriched in the *Blood microparticle* and *immunoglobulin complex* pathways [56]. The biological pathway *Blood microparticle* was identified as relevant to the DEGs in high-white and low-white count B-cells as well [57]. According to Huang *et al*. [58], cellular component enrichment analysis revealed that the DEGs distinguishing TP53 mutation from wild-type AML patients were significantly associated with the *Blood microparticle* pathway. The *Blood microparticle* pathway was also identified as being enriched with DEGs related to chronic lymphocytic leukemia [59].
- *Nitric-oxide synthase binding*
- According to Brandao *et al*. [60], AML patients exhibited elevated levels of nitric oxide synthase expression relative to controls.
- *Immune response*
- Fu *et al*. [61] discovered that upregulated leukemia-promoting genes were significantly enriched in biological processes such as cell activation involved in *Immune response, cytokine production, and leukocyte migration*. The differentially expressed probe sets that exhibit sex-specific variations in AML patients are significantly enriched in pathways related to *extracellular space, immune response, protein binding* [62]. Yang *et al*. [63] showed that the DEGs distinguishing high-risk from low-risk AML patients were predominantly associated with immune response signaling pathways. Deepak *et al*. [64] revealed that the network of DEGs in AML compared to normal samples was enriched in immune response-related processes.
- *Adaptive immune response*
- The GO term *Adaptive immune response* was highlighted as an enriched pathway for the differentially expressed long non-coding RNAs in AML [65].
- *Iumenal side of endoplasmic reticulum membrane*
- Hu *et al*. [66] identified the GO term *Iumenal side of endoplasmic reticulum membrane* as one of the top ten pathways associated with aberrant methylation DEGs in AML.
- *Antigen processing and presentation of exogenous peptide antigen via MHC class II*
- It was revealed that the enriched pathway for DEGs in common myeloid progenitor cells was the GO term *Antigen processing and presentation of exogenous peptide antigen via MHC class II* [67].

It can be seen through the literature that the identified biological pathways for genes linked the extracted the common edges in BALL, TALL and AML networks are key biological functions and may provide crucial clue for understanding AML mechanism.

From our results, we suggest that the suppression of ACTB could hold the key to comprehending the progression of acute leukemia. We also suggest that targeting the molecular interactions among the genes extracted from the shared edges, as displayed in Table 5, could offer pivotal insights for the prevention and treatment of acute leukemia.

We also compare the classifiers of various methods. The classifiers of GNmC, LA, KSVM and RF with 100 genes show 93% classification accuracies. Thus, we compare the classifiers of the methods for 100 genes. Table 7 shows the classified cell lines by the classifiers of methods.

**Table 7:** Comparison of classifiers with 100 genes.

TRUE label		GNmC	LA	KSVM	RF
sample5	BALL	BALL	BALL	BALL	BALL
sample7	BALL	BALL	BALL	BALL	BALL
sample8	BALL	BALL	BALL	BALL	BALL
sample9	TALL	TALL	TALL	TALL	TALL
sample10	TALL	TALL	TALL	TALL	TALL
sample24	BALL	BALL	BALL	BALL	BALL
sample25	BALL	BALL	BALL	BALL	BALL
sample29	AML	AML	AML	AML	AML
sample32	AML	AML	AML	AML	AML
sample38	AML	AML	AML	AML	AML
sample40	BALL	BALL	BALL	BALL	BALL
sample47	AML	BALL	BALL	BALL	BALL
sample48	BALL	BALL	BALL	BALL	BALL
sample54	AML	AML	AML	AML	AML
sample61	AML	AML	AML	AML	AML

As shown in Table 7, the methods that provide same accuracy (i.e., 93%.) construct same classifier. Although the methods show same classifiers for cell line classification, we can expect that our method provides more interpretable results for the classification, as our strategy provides information of not only crucial genes but also molecular interplays.

### Evaluations for multi-class cancer classification

We also evaluate our method for multi-class cancer classifications.

#### Mixed lineage leukemia dataset.

**Fig 6:**
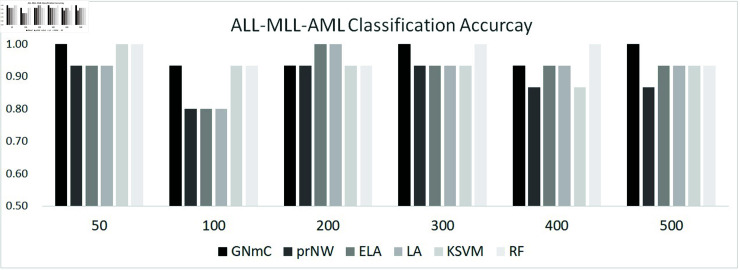
ALL-MLL-AML classification results.

We consider another popular acute leukemia data, called MLL dataset, for 12582 gene expression values for 72 peripheral blood or bone marrow samples consisting of 24 ALL, 20 MLL (mixed lineage leukemia) and 28 AML samples [68]. The MLL Leukemia dataset has been widely explored in research on multi-class cancer analysis and mult-classi classification [69, 70]. For each 24 ALL, 20 MLL and 28 AML samples, we generated the training and test dataset consist of 80% and 20% samples, respectively. Similar to the BALL-TALL-AML classification, we perform ALL-MLL-AML classification based on 50, 100, 200, 300, 400, and 500 genes with the highest variance in 72 samples. Figure 6 shows the classification results of ALL, MLL and AML samples. As shown in Figure 6, our method and RF show the outstanding results for the ALL-MLL-AML classification in overall. The proposed method (i.e., GNmC), KSVM and RF show the most effect classification accuracy (i.e., 100%, 93%, 100% and 100%) based on the models with 50 genes. On the other hand, the expression levels based classification by LA and ELA shows the perfect classification accuracy based on the models with 200 genes. Although some methods shows effective accuracies by classifier with 300, 400, 500 genes (i.e., GNmC and RF), the results implies that the larger number of features does not always provide the highest classification accuracy.

#### Lung cancer subtype classification.

**Fig 7:**
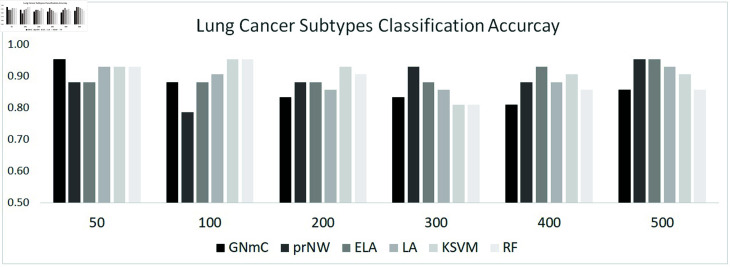
Lung cancer subtype classification results.

Our strategy is also applied to classification of not the leukemia cell lines but lung cancer subtypes. We used the publicly available CCLE expression dataset consisting of mRNA expression levels of 19,221 genes in 1,406 cell lines from the DepMap database (https://depmap.org/portal/). From 1,406 cell lines, we extract 206 cell lines that indicate “primary disease" as “Lung Cancer" (see Table 8). The training and test dataset are randomly selected from 80% and 20% of each type of cell lines, respectively. The classification accuracies are also evaluated by classifiers with 50, 100, 200, 300, 400, and 500 genes. The results of lung cancer subtype classification are given in Figure 7. In lung cancer subtype classification, almost methods (i.e., GNmC, LA, KSVM and RF) shows effective classification performances based on not the largest genes, while classifiers with the largest number of genes (i.e., 500) based on prNW and ELA shows the effective results. Our strategy also shows the most effective results based on the classifier with 50 genes. The results also implies that increasing number of features cannot always improve classification accuracies. We can expect through the results of various cancer classification that the proposed strategies will be a useful tool for multi-class classification of disease subtype.

**Table 8:** Subtypes of Lung cancer cell lines of CCLE expression levels dataset

Subtypes	Subtype	♯ cell lines
Otherwise	Carcinoid	1
	Mesothelioma	20
Non-Small Cell Lung Cancer	NSCLC, Adenocarcinoma	76
	NSCLC, Adenosquamous Carcinoma	4
	NSCLC, Large Cell Carcinoma	17
	NSCLC, Mucoepidermoid Carcinoma	1
	NSCLC, Squamous Cell Carcinoma	27
	NSCLC, unspecified	10
Small Cell Lung Cancer	SCLC	50

## Discussion

The mechanisms involved in disease are related to perturbations in complex molecular networks, rather than in a single gene, thus gene networks are crucial to understand complex mechanisms of disease [1]. The single gene-based analysis cannot provide comprehensive understanding of the disease mechanism. It implies that the expression-based classification cannot describe crucial molecular interplays to understand status of cell lines and/or cancer subtype. The crucialness of the network-based approaches has been demonstrated by many previous studies. Kim *et al*. [71] proposed a deep learning strategy for drug response prediction based on protein-protein interaction and demonstrated that the network-based prediction can reveal the subnetworks of genes that contribute to the drug response. Rapaport *et al*. [72] also proposed a classification methodology by incorporating the knowledge of the gene network a priori. Their strategy was applied to dataset of transcriptional response of irradiated and non-irradiated yeast colonies, and provided the accurate and interpretable discriminative model that may lead to new biological insights. Mi *et al*. [73] developed a disease classification model and demonstrated that the disease classification models based on gene network enable us to look at diseases in the viewpoint of commonalities in etiology and pathology.

In this study, we have introduced a novel computational strategy for gene network analysis and multi-class classification, i.e., method for gene regulatory network-based multi-class classification. The proposed GRN-multiClassifier aims to estimate the gene regulatory network while simultaneously minimizing both the error in gene network estimation and the negative log-likelihood linked to the multinomial logistic regression model. This indicates that the gene network obtained through our approach is the fine-tuned network designed for optimal multi-class classification. This enables us to achieve a biologically meaningful interpretation of gene network analysis and classification, as the estimated gene network is optimized to elucidate the cancer-related status of cell lines. We can expect through our results and the literature that the proposed network-based classifier will be a useful to biologically reliable and interpretable classification, because our method can explain the status and subtype of cancer cell lines based on the not only expression levels of genes but also their interplay.

To demonstrate the effectiveness of the proposed strategy, we conduct Monte Carlo simulations. The simulation results clearly show that the proposed GRN-multiClassifier outperforms methods relying on pre-estimated gene networks as well as methods that use gene expression levels for multi-class classification. Additionally, our strategy yields effective results in terms of feature selection for multinomial logistic regression and the selection of edges in network estimation.

We apply the GRN-multiClassifier to the classification of acute leukemia cell lines based on gene networks. Our approach demonstrates superior performance in classifying acute leukemia cell lines across all three distinct types. Through the interpretation of the optimized estimated networks for acute leukemia cell line classification, we identify essential markers and fundamental molecular interactions that play crucial roles in achieving accurate classification. Integrating information from prior research, the identified markers demonstrate substantial evidence that underlines their significance in mechanisms associated with acute leukemia. Our findings imply that ACTB may potentially play a pivotal role in the context of acute leukemia. Furthermore, the suppression of ACTB and the molecular interactions involving pairs such as “HBA1&HBB", ”HBB&HBA1", “IGKV1-5&IGHV4-31", “IGHV4-31&IGKV1-5", “HLA-DRA&CD74", and “A CTB&ACTB" might provide essential insights into unraveling the intricate mechanisms of acute leukemia. These insights could extend to implications for both preventive and therapeutic strategies.

Our strategy provides data-driven results for gene networks analysis, where the network is the optimized molecular interplays to explain status of cell lines. Although our method can estimate the optimized gene network for describing the diseases-related status of cell lines, the use of the known a-priory network can improve interpretability of the network-based classification results. That is, we consider the use of a known a-priory network related a specific disease (e.g., the networks involved in specific biological pathway in the known database) as an initial network in network-based classification, and then the known a-priory network is estimated to optimize for the specific disease status related classification. We consider the known a-priory network-based classification as one of the future works of this study and expect that the use of the known network can provide biologically interpretable and reliable results.

## Appendix

The first and second derivatives of the objective function of the GRN-multiClassifier in (13) with respect to $\theta_{g}$ are given by


$$\frac{\partial \ell \ell (B,\Theta )}{\partial \theta_{g}}$$

$$=\sum_{i=1}Z_{ig}x_{i}^{T}B-\sum_{i=1}^{n}\frac{\text{exp}(\theta_{g0}+x_{i}^{T}B\theta_{g})}{\sum_{g=1}^{G}\text{exp}(\theta_{g0}+x_{i}^{T}B\theta_{g})}(x_{i}^{T}B{)}^{T}$$

$$=\sum_{i=1}Z_{ig}x_{i}^{T}B-\sum_{i=1}^{n}\pi_{g}(x_{i},B)(x_{i}^{T}B{)}^{T}$$

$$=\sum_{i=1}\{Z_{ig}-\pi_{g}(x_{i},B)\}(x_{i}^{T}B{)}^{T}$$

$$=B^{T}X^{T}\Lambda_{g}1_{n}.$$

$$\frac{\partial^{2}\ell \ell (B,\Theta )}{\partial \theta_{g}\partial \theta_{g}^{T}}$$

$$=\;-\;\sum_{i=1}^{n}\;\frac{\text{exp}(\theta_{g0}+x_{i}^{T}B\theta_{g})\;\sum_{g=1}^{G}\text{exp}(\theta_{g0}+x_{i}^{T}B\theta_{g})\;-\;{\text{exp}(\theta_{g0}+x_{i}^{T}B\theta_{g})}^{2}}{\{\sum_{g=1}^{G}\text{exp}(\theta_{g0}+x_{i}^{T}B\theta_{g}){\}}^{2}}\;(x_{i}^{T}B{)}^{T}(x_{i}^{T}B)$$

$$=-\sum_{i=1}^{n}\pi_{g}(x_{i},B)\{1-\pi_{g}(x_{i},B)\}B^{T}x_{i}x_{i}^{T}B$$

$$=-B^{T}X^{T}\Pi_{g}(I_{n}-\Pi_{g})XB\mathrm{\backslash nonumber}$$

where $I_{n}$ is n×n identify matrix, $1_{n}=(1,...,1{)}^{T}$ is an *n*–dimensaional vector and $\Lambda_{g}$ and $\Pi_{g}$ are n×n diagonal matrices defined as


$$\Lambda_{g}=\text{diag}[Z_{1g}-\pi_{g}(x_{1},B),Z_{2g}-\pi_{g}(x_{2},B),...,Z_{ng}-\pi_{g}(x_{n},B)],$$

$$\Pi_{g}=\text{diag}[\pi_{g}(x_{1},B),\pi_{g}(x_{2},B),...,\pi_{g}(x_{n},B)].$$

Initiating from an initial value, we achieve a numerical solution by employing the subsequent update formula:


$$\theta_{g}^{\text{new}}=\theta_{g}^{\text{old}}-[E(\frac{\partial \ell \ell }{\partial \theta_{g}\partial^{T}\theta_{g}}){]}^{-1}\frac{\partial \ell \ell }{\partial \theta_{g}}.$$

The update formula is referred to as Fisher’s scoring algorithm, and the $(r\;+\;1{)}^{st}$ estimator $\theta_{g}^{\left(r+1\right)}$ is updated by


$$\theta_{g}^{\left(r+1\right)}=(\{B^{T}X^{T}\Pi_{g}^{\left(r\right)}(I_{n}-\Pi_{g}{)}^{\left(r\right)}XB{\}}^{-1}B^{T}X^{T}\Pi_{g}^{\left(r\right)}\{I_{n}-\Pi_{g}^{\left(r\right)}))k_{g}^{\left(r\right)},$$

where $k_{g}^{\left(r\right)}=XB\theta_{g}^{\left(r\right)}+\{\Pi_{g}^{\left(r\right)}\{I_{n}-\Pi_{g}^{\left(r\right)}){\}}^{-1}\Lambda_{g}1_{n}$ [14].

## References

[1] Ahmed KT, Park S, Jiang Q, Yeu Y, Hwang T, Zhang W. Network-based drug sensitivity prediction. BMC Med Genomics. 2020;13(Suppl 11):193. doi: 10.1186/s12920-020-00829-3 33371891 PMC7771088

[2] Daoud M, Mayo M. A survey of neural network-based cancer prediction models from microarray data. Artif Intell Med. 2019;97:204–14. doi: 10.1016/j.artmed.2019.01.006 30797633

[3] Cheng F, Kovács IA, Barabási A-L. Network-based prediction of drug combinations. Nat Commun. 2019;10(1):1197. doi: 10.1038/s41467-019-09186-x 30867426 PMC6416394

[4] Fout A, Byrd J, Shariat B, Ben-Hur A. Protein interface prediction using graph convolutional networks. In: NIPS’17: Proceedings of the 31st International Conference on Neural Information Processing Systems, 2017, pp. 6533–42.

[5] Kamada M, Takagi A, Kojima R, Tanaka Y, Nakatsui M, et al. Network-based pathogenicity prediction for variants of uncertain significance. bioRxiv. preprint. 2021. [cited August 14, 2021]. doi: 10.1101/2021.07.15.452566

[6] Velickovic P, Cucurull G, Casanova A, Romero A, Liò P, et al. Graph Attention Networks. arXiv. preprint. 2018. arXiv:1710.10903 [submitted February 4, 2018]. Available from: https://arxiv.org/abs/1710.10903.

[7] Chatrabgoun H, Soltanian AR, Mahjub H, Bahreini F. Learning gene regulatory networks using gaussian process emulator and graphical LASSO. J Bioinform Comput Biol. 2021;19(3):2150007. doi: 10.1142/S0219720021500074 33930997

[8] Imoto S, Kim S, Goto T, Miyano S, Aburatani S, Tashiro K, et al. Bayesian network and nonparametric heteroscedastic regression for nonlinear modeling of genetic network. J Bioinform Comput Biol. 2003;1(2):231–52. doi: 10.1142/s0219720003000071 15290771

[9] Park H, Imoto S, Miyano S. Gene Regulatory Network-Classifier: Gene Regulatory Network-Based Classifier and Its Applications to Gastric Cancer Drug (5-Fluorouracil) Marker Identification. J Comput Biol. 2023;30(2):223–43. doi: 10.1089/cmb.2022.0181 36450117

[10] Golub TR, Slonim DK, Tamayo P, Huard C, Gaasenbeek M, Mesirov JP, et al. Molecular classification of cancer: class discovery and class prediction by gene expression monitoring. Science. 1999;286(5439):531–7. doi: 10.1126/science.286.5439.531 10521349

[11] Hoerl AE, Kennard RW. Ridge regression: biased estimation for nonorthogonal problems. Techonometrics. 1970;12:55–67. doi: 10.2307/1271436

[12] Tibshirani R. Regression Shrinkage and Selection Via the Lasso. Journal of the Royal Statistical Society Series B: Statistical Methodology. 1996;58(1):267–88. doi: 10.1111/j.2517-6161.1996.tb02080.x

[13] Zou H, Hastie T. Regularization and Variable Selection Via the Elastic Net. Journal of the Royal Statistical Society Series B: Statistical Methodology. 2005;67(2):301–20. doi: 10.1111/j.1467-9868.2005.00503.x

[14] Konishi S, Kitagawa G. Information criteria and statistical modeling. New York, USA: Springer; 2008.

[15] Yu D, Lim J, Wang X, Liang F, Xiao G. Enhanced construction of gene regulatory networks using hub gene information. BMC Bioinformatics. 2017;18(1):186. doi: 10.1186/s12859-017-1576-1 28335719 PMC5364645

[16] Li C, Li H. Network-constrained regularization and variable selection for analysis of genomic data. Bioinformatics. 2008;24(9):1175–82. doi: 10.1093/bioinformatics/btn081 18310618

[17] Sun H, Lin W, Feng R, Li H. Network-regularized high-dimensional cox regression for analysis of genomic data. Stat Sin. 2014;24(3):1433–59. doi: 10.5705/ss.2012.317 26316678 PMC4549005

[18] Tibshirani R, Saunders M, Rosset S, Zhu J, Knight K. Sparsity and Smoothness Via the Fused Lasso. J Roy Stat Soc Ser B. 2005;67(1):91–108. doi: 10.1111/j.1467-9868.2005.00490.x

[19] Zou H, Hastie T, Tibshirani R. On the “degrees of freedom” of the lasso. Ann Statist. 2007;35(5). doi: 10.1214/009053607000000127

[20] Liu X, Tang H, Ding Y, Yan D. Investigating the performance of machine learning models combined with different feature selection methods to estimate the energy consumption of buildings. Energy and Buildings. 2022;273:112408. doi: 10.1016/j.enbuild.2022.112408

[21] Tan Y, Shi L, Tong W, Wang C. Multi-class cancer classification by total principal component regression (TPCR) using microarray gene expression data. Nucleic Acids Res. 2005;33(1):56–65. doi: 10.1093/nar/gki144 15640445 PMC546133

[22] Bicciato S, Luchini A, Di Bello C. PCA disjoint models for multiclass cancer analysis using gene expression data. Bioinformatics. 2003;19(5):571–8. doi: 10.1093/bioinformatics/btg051 12651714

[23] Wang L, Li J, Liu J, Chang M. RAMRSGL: A Robust Adaptive Multinomial Regression Model for Multicancer Classification. Comput Math Methods Med. 2021;2021:5584684. doi: 10.1155/2021/5584684 34122617 PMC8172296

[24] Lee Y, Lee C-K. Classification of multiple cancer types by multicategory support vector machines using gene expression data. Bioinformatics. 2003;19(9):1132–9. doi: 10.1093/bioinformatics/btg102 12801874

[25] Zhao Y, Si Y, Zhang W, Huang W, Wang R. Elane is highly expressed in leukemia patients and predicts poor survival. Int J Clin Exp Med. 2019;12(4):3153–60.

[26] Rotulo GA, Beaupain B, Rialland F, Paillard C, Nachit O, Galambrun C, et al. HSCT may lower leukemia risk in ELANE neutropenia: a before-after study from the French Severe Congenital Neutropenia Registry. Bone Marrow Transplant. 2020;55(8):1614–22. doi: 10.1038/s41409-020-0800-1 31992846 PMC7091645

[27] Jiang L, Li X-P, Dai Y-T, Chen B, Weng X-Q, Xiong S-M, et al. Multidimensional study of the heterogeneity of leukemia cells in t(8;21) acute myelogenous leukemia identifies the subtype with poor outcome. Proc Natl Acad Sci U S A. 2020;117(33):20117–26. doi: 10.1073/pnas.2003900117 32747558 PMC7443908

[28] Li Y, Cheng J, Li Y, Jiang Y, Ma J, Li Q, et al. CXCL8 is associated with the recurrence of patients with acute myeloid leukemia and cell proliferation in leukemia cell lines. Biochem Biophys Res Commun. 2018;499(3):524–30. doi: 10.1016/j.bbrc.2018.03.181 29596823

[29] Cheng J, Li Y, Liu S, Jiang Y, Ma J, Wan L, et al. CXCL8 derived from mesenchymal stromal cells supports survival and proliferation of acute myeloid leukemia cells through the PI3K/AKT pathway. FASEB J. 2019;33(4):4755–64. doi: 10.1096/fj.201801931R 30592634

[30] Soltani S, Mozhgani S-H, Siri G, Emadi MS, Rahimi Foroushani A, Jazayeri SM, et al. High expression of inflammatory cytokines and chemokines in human t-lymphotropic virus 1-associated adult t-cell leukemia/lymphoma. Jundishapur J Microbiol. 2022;15(10). doi: 10.5812/jjm-132348

[31] Chen AH, Tsau Y-W, Lin C-H. Novel methods to identify biologically relevant genes for leukemia and prostate cancer from gene expression profiles. BMC Genomics. 2010;11:274. doi: 10.1186/1471-2164-11-274 20433712 PMC2873479

[32] Sun Y, Dong L-J, Tian F, Wang S-Q, Jia Z-L, Huang J, et al. Identification of acute leukemia-specific genes from leukemia recipient/sibling donor pairs by distinguishing study with oligonucleotide microarrays. Zhongguo Shi Yan Xue Ye Xue Za Zhi. 2004;12(4):450–4. 15363129

[33] Koo J-Y, Sohn I, Kim S, Lee JW. Structured polychotomous machine diagnosis of multiple cancer types using gene expression. Bioinformatics. 2006;22(8):950–8. doi: 10.1093/bioinformatics/btl029 16452113

[24] Yuita H, López-Moyado IF, Jeong H, Cheng AX, Scott-Browne J, An J, et al. Inducible disruption of Tet genes results in myeloid malignancy, readthrough transcription, and a heterochromatin-to-euchromatin switch. Proc Natl Acad Sci U S A. 2023;120(6):e2214824120. doi: 10.1073/pnas.2214824120 37406303 PMC9963276

[35] Wang Y, Li X, Ruiz R. Weighted general group lasso for gene selection in cancer classification. IEEE Trans Cybern. 2019;49(8):2860–73. doi: 10.1109/TCYB.2018.2829811 29993764

[36] Itonaga H, Imanishi D, Wong Y-F, Sato S, Ando K, Sawayama Y, et al. Expression of myeloperoxidase in acute myeloid leukemia blasts mirrors the distinct DNA methylation pattern involving the downregulation of DNA methyltransferase DNMT3B. Leukemia. 2014;28(7):1459–66. doi: 10.1038/leu.2014.15 24457336

[37] Krajinovic M, Sinnett H, Richer C, Labuda D, Sinnett D. Role of NQO1, MPO and CYP2E1 genetic polymorphisms in the susceptibility to childhood acute lymphoblastic leukemia. Int J Cancer. 2002;97(2):230–6. doi: 10.1002/ijc.1589 11774269

[38] Oberley MJ, Li S, Orgel E, Phei Wee C, Hagiya A, O’Gorman MRG. Clinical significance of isolated myeloperoxidase expression in pediatric B-Lymphoblastic Leukemia. Am J Clin Pathol. 2017;147(4):374–81. doi: 10.1093/ajcp/aqx021 28340210

[39] Kim YR, Eom JI, Kim SJ, Jeung HK, Cheong J-W, Kim JS, et al. Myeloperoxidase expression as a potential determinant of parthenolide-induced apoptosis in leukemia bulk and leukemia stem cells. J Pharmacol Exp Ther. 2010;335(2):389–400. doi: 10.1124/jpet.110.169367 20699435

[40] Zaki SR, Austin GE, Swan D, Srinivasan A, Ragab AH, Chan WC. Human myeloperoxidase gene expression in acute leukemia. Blood. 1989;74(6):2096–102. 2553160

[41] Kumari P, LingappaKavitha B, Obula Reddy C, Mangalagowri M, Madhumathi DS, Mahadeva Prasad M, et al. A rare cytogenetic presentation of acute myeloid leukemia (AML-M2). Acta Med Iran. 2012;50(12):827–30. 23456526

[42] Tobe H, Ruether BA, Jerry LM, Tamaoki T. Mythylation of human HLA-D/DR genes: derangement in chronic lymphocytic leukemia. Cancer Biochem Biophys. 1986;8(4):313–26. 3492264

[43] Dorak MT, Lawson T, Machulla HK, Darke C, Mills KI, Burnett AK. Unravelling an HLA-DR association in childhood acute lymphoblastic leukemia. Blood. 1999;94(2):694–700. 10397736

[44] Morrison BA, Ucisik-Akkaya E, Flores H, Alaez C, Gorodezky C, Dorak MT. Multiple sclerosis risk markers in HLA-DRA, HLA-C, and IFNG genes are associated with sex-specific childhood leukemia risk. Autoimmunity. 2010;43(8):690–7. doi: 10.3109/08916930903567492 21067287

[45] Le Q, Tang T, Leonti A, Castro S, McKay CN, Perkins L, et al. Preclinical studies targeting CD74 with STRO-001 antibody-drug conjugate in acute leukemia. Blood Adv. 2023;7(9):1666–70. doi: 10.1182/bloodadvances.2022008303 36595452 PMC10182275

[46] Aggarwal M, Villuendas R, Gomez G, Rodriguez-Pinilla SM, Sanchez-Beato M, Alvarez D, et al. TCL1A expression delineates biological and clinical variability in B-cell lymphoma. Mod Pathol. 2009;22(2):206–15. doi: 10.1038/modpathol.2008.148 18820675

[47] Yokohama A, Saitoh A, Nakahashi H, Mitsui T, Koiso H, Kim Y, et al. TCL1A gene involvement in T-cell prolymphocytic leukemia in Japanese patients. Int J Hematol. 2012;95(1):77–85. doi: 10.1007/s12185-011-0986-5 22189846

[48] Garza-Veloz I, Martinez-Fierro ML, Jaime-Perez JC, Carrillo-Sanchez K, Ramos-Del Hoyo MG, Lugo-Trampe A, et al. Identification of differentially expressed genes associated with prognosis of B acute lymphoblastic leukemia. Dis Markers. 2015;2015:828145. doi: 10.1155/2015/828145 25802479 PMC4354728

[49] Te Kronnie G, Bicciato S, Franceschini L, Accordi B, Dellíorto MC, Rinaldi A, et al. Validation by RQ-PCR and flow cytometry of alpha-defensin1-3 (DEFA1-3) overexpression in relapsed and refractory acute lymphoblastic leukemia. Oncol Rep. 2006;15(2):341–6. 16391852

[50] van der Velden VHJ, Brüggemann M, Hoogeveen PG, de Bie M, Hart PG, Raff T, et al. TCRB gene rearrangements in childhood and adult precursor-B-ALL: frequency, applicability as MRD-PCR target, and stability between diagnosis and relapse. Leukemia. 2004;18(12):1971–80. doi: 10.1038/sj.leu.2403505 15470492

[51] de Kok JB, Roelofs RW, Giesendorf BA, Pennings JL, Waas ET, Feuth T, et al. Normalization of gene expression measurements in tumor tissues: comparison of 13 endogenous control genes. Lab Invest. 2005;85(1):154–9. doi: 10.1038/labinvest.3700208 15543203

[52] Braoudaki M, Lambrou GI, Vougas K, Karamolegou K, Tsangaris GT, Tzortzatou-Stathopoulou F. Protein biomarkers distinguish between high- and low-risk pediatric acute lymphoblastic leukemia in a tissue specific manner. J Hematol Oncol. 2013;6:52. doi: 10.1186/1756-8722-6-52 23849470 PMC3717072

[53] Fernández-Torres J, Flores-Jiménez D, Arroyo-Pérez A, Granados J, López-Reyes A. HLA-B*40 allele plays a role in the development of acute leukemia in Mexican population: a case-control study. Biomed Res Int. 2013;2013:705862. doi: 10.1155/2013/705862 24364037 PMC3858009

[54] Balas A, Planelles D, Goterris R, Rodríguez-Cebriá M, Vicario JL. Somatic mutation in the two HLA-B genes of a patient with acute myelogenous leukemia. HLA. 2019;94(4):360–4. doi: 10.1111/tan.13640 31338977

[55] Mishra VC, Deshpande T, Gupta N, Dorwal P, Chandra D, Raina V, et al. Frequency analysis of HLA-B allele in leukemia patients from a North Indian population: A case-control study. Meta Gene. 2021;27:100842. doi: 10.1016/j.mgene.2020.100842

[56] Wang Q, Yue C, Liu Q, Che X. Exploration of differentially expressed mRNAs and miRNAs for pediatric acute myeloid leukemia. Front Genet. 2022;13:865111. doi: 10.3389/fgene.2022.865111 36160019 PMC9499657

[57] Kumar A, Bhushan R, Dubey PK, Tilak V, Gupta V, et al. In silico analysis of genes and pathways related to acute myeloid leukemia presenting leukopenia. Res Sqaure. 2021. doi: 10.21203/rs.3.rs-1019863/v2

[58] Huang R, Liao X, Li Q. Identification of key pathways and genes in TP53 mutation acute myeloid leukemia: evidence from bioinformatics analysis. Onco Targets Ther. 2017;11:163–73. doi: 10.2147/OTT.S156003 29343974 PMC5749383

[59] Gao C, Zhou C, Zhuang J, Liu L, Wei J, Liu C, et al. Identification of key candidate genes and miRNA‑mRNA target pairs in chronic lymphocytic leukemia by integrated bioinformatics analysis. Mol Med Rep. 2019;19(1):362–74. doi: 10.3892/mmr.2018.9636 30431072 PMC6297738

[60] Brandão MM, Soares E, Salles TS, Saad ST. Expression of inducible nitric oxide synthase is increased in acute myeloid leukaemia. Acta Haematol. 2001;106(3):95–9. doi: 10.1159/000046596 11713373

[61] Fu D, Zhang B, Wu S, Feng J, Jiang H. Molecular subtyping of acute myeloid leukemia through ferroptosis signatures predicts prognosis and deciphers the immune microenvironment. Front Cell Dev Biol. 2023;11:1207642. doi: 10.3389/fcell.2023.1207642 37691822 PMC10483833

[62] Roushangar R, Mias GI. Multi-study reanalysis of 2,213 acute myeloid leukemia patients reveals age- and sex-dependent gene expression signatures. Sci Rep. 2019;9(1):12413. doi: 10.1038/s41598-019-48872-0 31455838 PMC6712049

[63] Yang Y, Yang Y, Liu J, Zeng Y, Guo Q, Guo J, et al. Establishment and validation of a carbohydrate metabolism-related gene signature for prognostic model and immune response in acute myeloid leukemia. Front Immunol. 2022;13:1038570. doi: 10.3389/fimmu.2022.1038570 36544784 PMC9761472

[64] Deepak Shyl ES, Malgija B, Iniyan AM, Mahendran R, Prakash Vincent SG. Mining of transcriptome identifies CD109 and LRP12 as possible biomarkers and deregulation mechanism of T cell receptor pathway in Acute Myeloid Leukemia. Heliyon. 2022;8(10):e11123. doi: 10.1016/j.heliyon.2022.e11123 36299526 PMC9589179

[65] Feng Y, Shen Y, Chen H, Wang X, Zhang R, Peng Y, et al. Expression profile analysis of long non-coding RNA in acute myeloid leukemia by microarray and bioinformatics. Cancer Sci. 2018;109(2):340–53. doi: 10.1111/cas.13465 29220122 PMC5797823

[66] Hu L, Gao Y, Shi Z, Liu Y, Zhao J, Xiao Z, et al. DNA methylation-based prognostic biomarkers of acute myeloid leukemia patients. Ann Transl Med. 2019;7(23):737. doi: 10.21037/atm.2019.11.122 32042753 PMC6989983

[67] Shirai CL, Ley JN, White BS, Kim S, Tibbitts J, Shao J, et al. Mutant U2AF1 Expression Alters Hematopoiesis and Pre-mRNA Splicing In Vivo. Cancer Cell. 2015;27(5):631–43. doi: 10.1016/j.ccell.2015.04.008 25965570 PMC4430854

[68] Armstrong SA, Staunton JE, Silverman LB, Pieters R, den Boer ML, Minden MD, et al. MLL translocations specify a distinct gene expression profile that distinguishes a unique leukemia. Nat Genet. 2002;30(1):41–7. doi: 10.1038/ng765 11731795

[69] Huang L-T. An integrated method for cancer classification and rule extraction from microarray data. J Biomed Sci. 2009;16(1):25. doi: 10.1186/1423-0127-16-25 19272192 PMC2653531

[70] Liu B, Cui Q, Jiang T, Ma S. A combinational feature selection and ensemble neural network method for classification of gene expression data. BMC Bioinformatics. 2004;5:136. doi: 10.1186/1471-2105-5-136 15450124 PMC522806

[71] Nguyen T, Nguyen GTT, Nguyen T, Le D-H. Graph Convolutional Networks for Drug Response Prediction. IEEE/ACM Trans Comput Biol Bioinform. 2022;19(1):146–54. doi: 10.1109/TCBB.2021.3060430 33606633

[72] Rapaport F, Zinovyev A, Dutreix M, Barillot E, Vert J-P. Classification of microarray data using gene networks. BMC Bioinformatics. 2007;8:35. doi: 10.1186/1471-2105-8-35 17270037 PMC1797191

[73] Mi Z, Guo B, Yin Z, Li J, Zheng Z. Disease classification via gene network integrating modules and pathways. R Soc Open Sci. 2019;6(7):190214. doi: 10.1098/rsos.190214 31417727 PMC6689581

